# Prostaglandin E_2_ Boosts the Hyaluronan-Mediated Increase in Inflammatory Response to Lipopolysaccharide by Enhancing Lyve1 Expression

**DOI:** 10.3390/biology12111441

**Published:** 2023-11-16

**Authors:** Pauline Hog, Silvia Kuntschar, Peter Rappl, Arnaud Huard, Andreas Weigert, Bernhard Brüne, Tobias Schmid

**Affiliations:** 1 Institute of Biochemistry I, Faculty of Medicine, Goethe University Frankfurt, 60590 Frankfurt, Germany; 2 German Cancer Consortium (DKTK), Partner Site Frankfurt, 60590 Frankfurt, Germany; 3 Frankfurt Cancer Institute, Goethe University Frankfurt, 60596 Frankfurt, Germany; 4 Fraunhofer Institute for Translational Medicine and Pharmacology, 60596 Frankfurt, Germany

**Keywords:** macrophage, inflammation, resolution, hyaluronic acid, peritonitis

## Abstract

**Simple Summary:**

Inflammatory reactions provide a crucial defense mechanism against invading pathogens by generating a highly reactive environment. To limit tissue damage due to ongoing inflammation, resolution of inflammation is a tightly regulated process, which is orchestrated amongst other cell types by macrophages. While numerous functional macrophage phenotypes have been described, there is little information on how exactly different subtypes contribute to the resolution of inflammation. In the present study, we observed that expression of hyaluronan receptor Lyve1 in macrophages correlates with efficient resolution of inflammation. We further identified prostaglandin E_2_/EP2 receptor-mediated signaling to enhance Lyve1 expression in macrophages, contributing, as a consequence, to the sensitization of macrophages to synergistic inflammatory stimulation with lipopolysaccharide and the Lyve1 ligand low-molecular-weight hyaluronan. We thus propose that Lyve1-expressing macrophages are an important macrophage subpopulation able to integrate extracellular matrix-derived signals with pathogenic, inflammatory stimuli.

**Abstract:**

Macrophages are a highly versatile and heterogenic group of immune cells, known for their involvement in inflammatory reactions. However, our knowledge about distinct subpopulations of macrophages and their specific contribution to the resolution of inflammation remains incomplete. We have previously shown, in an in vivo peritonitis model, that inhibition of the synthesis of the pro-inflammatory lipid mediator prostaglandin E_2_ (PGE_2_) attenuates efficient resolution of inflammation. PGE_2_ levels during later stages of the inflammatory process further correlate with expression of the hyaluronan (HA) receptor Lyve1 in peritoneal macrophages. In the present study, we therefore aimed to understand if PGE_2_ might contribute to the regulation of Lyve1 and how this might impact inflammatory responses. In line with our in vivo findings, PGE_2_ synergized with dexamethasone to enhance *Lyve1* expression in bone marrow-derived macrophages, while expression of the predominant hyaluronan receptor *CD44* remained unaltered. PGE_2_-mediated *Lyve1* upregulation was strictly dependent on PGE_2_ receptor EP2 signaling. While PGE_2_/dexamethasone-treated macrophages, despite their enhanced *Lyve1* expression, did not show inflammatory responses upon stimulation with low (LMW) or high-molecular-weight hyaluronan (HMW)-HA, they were sensitized towards LMW-HA-dependent augmentation of lipopolysaccharide (LPS)-induced inflammatory responses. Thus, Lyve1-expressing macrophages emerged as a subpopulation of macrophages integrating inflammatory stimuli with extracellular matrix-derived signals.

## 1. Introduction

Inflammatory reactions are elicited by pathogens, such as viruses, bacteria, or fungi, to eliminate the underlying harmful stimulus [1,2]. Inflammatory conditions at the same time also affect cells within the local environment, also putting healthy cells at risk. To prevent unnecessary damage due to overshooting or chronic inflammation, underlying processes are tightly regulated with respect to both the degree of inflammation, but also its timely resolution. Resolution of inflammation, which is considered to start already early during the inflammatory phase, comprises termination of inflammatory processes and also the environmental and cellular return to a homeostatic state [3,4,5,6]. Interestingly, macrophages are known to contribute to the initiation and maintenance of inflammatory reactions, but are also critical for a successful resolution [4,7]. The key role of macrophages for all phases of inflammation underlines their high functional plasticity. Specifically, during early inflammation, macrophages acquire a pro-inflammatory phenotype supporting the elimination of pathogens [8], whereas at later stages anti-inflammatory as well as pro-resolving and wound healing macrophage phenotypes have been characterized [9,10]. The resolution phenotype is induced by pro-resolving mediators, including lipid (also described as specialized pro-resolving mediators (SPM)) and protein factors (e.g., transforming growth factor β) [8,11].

Immune cell populations and activation states are commonly classified by the expression of specific surface markers [12,13]. In macrophages, such surface marker profiles allow for the determination of the origin (e.g., tissue-resident vs. infiltrating), and the differentiation state, but also for the characterization of different polarization states [14,15]. Here, we specifically focused on a subpopulation of macrophages expressing the lymphatic vessel endothelial receptor 1 (Lyve1), a receptor for the glycosaminoglycan hyaluronan (hyaluronic acid; HA) [16]. Hyaluronan is broken down into smaller fragments during inflammation and was proposed to act as damage-associated molecular pattern (DAMP) [17,18]. Moreover, depending on the size of the resulting fragments, hyaluronan has been shown to act either pro- or anti-inflammatorily [18,19,20,21]. Lyve1-positive macrophages were recently described as a subpopulation of tissue-resident macrophages [22], linked to matrix-remodeling [23] and tumor-associated functions [24]. Lyve1-overexpressing macrophages were further shown to be enriched in non-inflammatory femoral plaques [25]. Here, we asked if Lyve1 might be relevant during the resolution of inflammation and, if so, how it might affect inflammatory responses.

## 2. Materials and Methods

### 2.1. Chemicals

All chemicals were purchased from Thermo Fisher Scientific GmbH (Dreieich, Germany), if not indicated otherwise. EP receptor antagonists came from Cayman Chemicals (Ann Abor, MI, USA). Primers were ordered from Biomers (Ulm, Germany).

### 2.2. Cell Culture

Bone marrow-derived macrophages (BMDM) from wildtype C57BL/6 mice were differentiated for 5 days using 20 ng/mL macrophage colony-stimulating factor (M-CSF) and 20 ng/mL granulocyte-macrophage colony-stimulating factor (GM-CSF) (Immunotools, Friesoythe, Germany) in Dulbecco’s Modified Eagle’s Medium (DMEM) containing 100 U/mL penicillin, 100 µg/mL streptomycin, and 10% fetal calf serum (FCS; Capricorn Scientific GmbH, Ebsdorfergrund, Germany). After differentiation, BMDM were stimulated with 100 ng/mL dexamethasone (Sigma-Aldrich, St. Louis, MO, USA) and 250 ng/mL prostaglandin E_2_ (PGE_2_) (Cayman Chemical, Ann Arbor, MI, USA) for 48 h in the absence of M-CSF/GM-CSF. Media were obtained from Thermo Fisher Scientific. All other cell culture supplements came from Sigma-Aldrich, if not indicated otherwise.

### 2.3. RNA Analyses

Total RNA from BMDM was isolated using TRIzol reagent (Thermo Fisher Scientific) in accordance with the manufacturer’s instructions. RNA was quantified on a Nanodrop instrument (PeqLab, Erlangen, Germany), reverse transcribed using Maxima First Strand cDNA synthesis kits, and gene expression was analyzed on QuantStudio^®^ 3 and 5 real-time PCR instruments using PowerUp SYBR Green Mix (all Thermo Fisher Scientific) with gene-specific primers (see Table 1).

### 2.4. Zymosan-Induced Peritonitis

To induce a transient peritonitis, 8–12-week-old female mice received an intraperitoneal injection of 5 mg/kg of body weight zymosan A (Sigma-Aldrich). One day after zymosan injection, the mice were randomly selected to receive either the mPGES-1 inhibitor compound III (CIII) (20 mg/kg) (kindly provided by P.-J. Jakobsson) or an appropriate vehicle control (1% Tween-80, 0.5% Carboxymethyl cellulose, 0.9% NaCl solution). Peritoneal immune infiltrates were obtained by flushing the peritoneal cavity with 3 mL of phosphate-buffer saline (PBS) and collecting the resulting peritoneal lavages for FACS analyses. All animal experiments followed the guidelines of the Hessian animal care and use committee (approval number: FU/1211).

### 2.5. FACS Analyses and Sorting

Single-cell suspensions of the peritoneal lavages were incubated with FcR-blocking reagent (Miltenyi Biotec, Bergisch Gladbach, Germany) for 15 min, before staining with an antibody mix for 20 min in the dark at 4 °C. Staining was performed in PBS containing 0.5% bovine serum albumin (BSA). F4/80^hi^ macrophages were sorted using a FACS ARIA III cell sorter (BD Biosciences, Heidelberg, Germany) [26]. In addition, Lyve1 antibody (ThermoFisher 53-0443-82) was added to determine Lyve1 surface expression.

### 2.6. RNA Sequencing

Total RNA was isolated from macrophages using the RNeasy Micro Kit (Qiagen, Hilden, Germany) according to the manufacturer’s instructions. RNA concentration was determined with a Qubit HS RNA Assay Kit (Thermo Fisher Scientific) and RNA integrity was analyzed on an Agilent 2100 Bioanalyzer using an RNA 6000 Pico Chip (Agilent Technologies, Waldbronn, Germany). Sequencing libraries were prepared using the QuantSeq 3′ fwd mRNA-Seq Library Prep Kit (Lexogen, Vienna, Austria). Quantity and quality of the cDNA libraries were evaluated by Qubit dsDNA HS Assay Kit (Thermo Fisher Scientific) and Agilent DNA High Sensitivity DNA Chip (Agilent Technologies), respectively. Libraries were sequenced (single end, 75 cycles) using a High Output Kit v2 on a NextSeq 500 sequencer (Illumina, San Diego, CA, USA). The data were analyzed using the Bluebee QuantSeq FWD Data Analysis Pipeline. Sequencing data have been deposited under GEO accession number GSE164364 [26].

### 2.7. Statistics

The data were analyzed using one-way ANOVA with Tukey’s post hoc test or a two-tailed paired or unpaired Student’s *t*-test using GraphPad Prism 10.0.2.

## 3. Results

### 3.1. Inhibition of mPGES-1 Reduces Lyve1 Expression in Macrophages during Resolution of Inflammation

**Figure 1:**
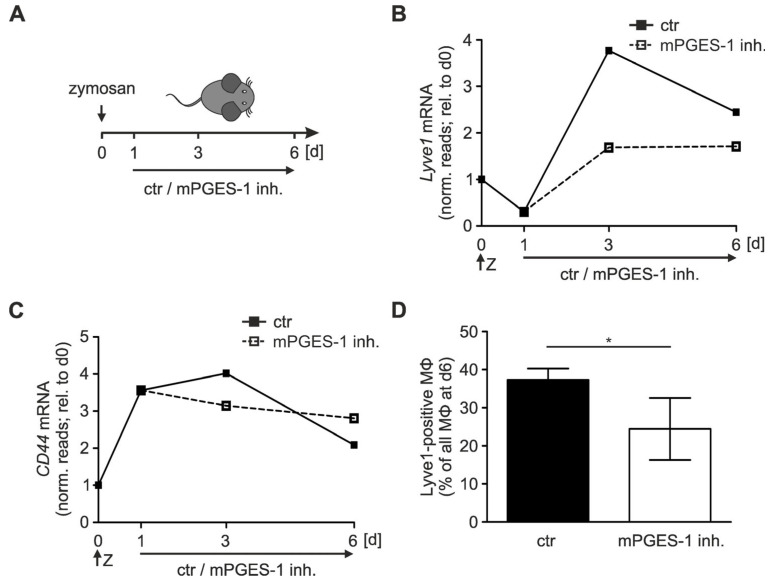
mPGES-1 inhibition attenuates *Lyve1* expression in a zymosan-induced peritonitis model. (**A**) Peritonitis was induced in C57/BL6 mice by i.p. injection of 5 mg/kg zymosan (Z). Starting 24 h after zymosan injection, the mPGES-1 inhibitor CIII (25 mg/kg) or the appropriate vehicle control were applied i.p. daily, to study the role of PGE_2_ in the resolution of inflammation. (**B**) *Lyve1* and (**C**) *CD44* mRNA expression in macrophages (MΦ) isolated from the peritoneum was assessed by RNA-seq at days 0, 1, 3, and 6. Data are given as mean library-normalized read counts relative to day 0. (**D**) The percentage of MΦ presenting Lyve1 at the surface relative to all MΦ (F4/80^hi^, MERTK^hi^) was determined by FACS analysis at day 6. Data are presented as mean ± SEM (*n* > 7; * *p* < 0.05).

We previously showed that, in the self-limiting zymosan-induced peritonitis model (5 mg/kg zymosan, i.p.), a reduction in prostaglandin E_2_ (PGE_2_) levels with the microsomal PGE_2_ synthase 1 (mPGES-1) inhibitor compound III (25 mg/kg) selectively, during the resolution phase (Figure 1A), resulted in inefficient resolution of inflammation [26]. Specifically, we observed that CX3CL1/CX3CR1-dependent retention of activated myeloid cells within the inflammatory environment upon mPGES-1 inhibition contributed to sustained inflammation. mRNA sequencing of macrophages isolated from peritoneal lavages further revealed that after a marked reduction at the peak of the inflammatory phase (day 1), *lymphatic vessel endothelial hyaluronan receptor 1* (*Lyve1*) mRNA expression markedly increased during resolution, especially at early resolution (day 3) (Figure 1B).

A much less pronounced increase in *Lyve1* mRNA was observed in macrophages isolated from mPGES-1-inhibited mice. As Lyve1 is a receptor for hyaluronan (hyaluronic acid, HA), we further evaluated expression of its paralog *cluster of differentiation 44* (*CD44*). In contrast to *Lyve1*, *CD44* mRNA already increased during the inflammatory phase and appeared to be unaffected by mPGES-1 inhibition during the resolution phase (Figure 1C). In line with the observed lower *Lyve1* mRNA expression in the mPGES-1-inhibited peritonitis setting, fewer Lyve1-positive macrophages were present in the peritoneal cavity, when PGE_2_ synthesis was blocked and resolution was attenuated (Figure 1D).

Thus, expression of the alternative HA receptor Lyve1 correlated with reduced PGE_2_ levels during resolution of inflammation in vivo, while no such association was observed for the predominant HA receptor CD44.

### 3.2. EP2 Signaling Enhances Lyve1 Expression

**Figure 2:**
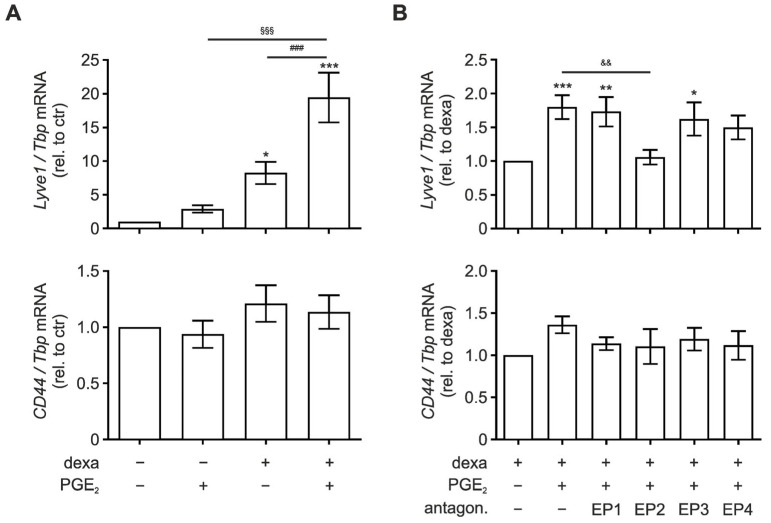
PGE_2_ enhances dexamethasone-induced *Lyve1* expression. Bone marrow-derived macrophages (BMDM) were differentiated for 5 days before stimulation for 48 h with (**A**) dexamethasone (dexa; 100 ng/mL), PGE_2_ (250 ng/mL), or a combination of dexa and PGE_2_. (**B**) To determine the role of the PGE_2_ receptors EP1-4, BMDM were separately pre-incubated with selective antagonists for EP1 (ONO 8130), EP2 (PF-04418948), EP3 (L-798,106), or EP4 (ONO AE3 208) (1 µM each) 30 min before stimulation with dexa and PGE_2_ for 48 h. mRNA expression of *Lyve1* (*upper panels*) and *CD44* (*lower panels*) was determined by RT-qPCR analysis. Data are normalized to *Tbp* (*TATA-box binding protein*) and presented relative to corresponding controls as mean ± SEM (*n* > 12; * *p* < 0.05; ** *p* < 0.01; *** *p* < 0.001; compared to respective controls (* untreated, ^#^ dexa, ^§^ PGE2, or ^&^ dexa + PGE2 stimulated)).

To assess how Lyve1 expression might be regulated, we stimulated bone marrow-derived macrophages (BMDM) in vitro with dexamethasone (dexa; 100 ng/mL), previously reported to induce *Lyve1* expression [27], PGE_2_ (250 ng/mL), or a combination of both for 48 h. In accordance with previous reports dexamethasone significantly induced *Lyve1* expression (Figure 2A, *upper panel*). Moreover, while PGE_2_ alone only slightly induced *Lyve1* expression, it markedly enhanced the dexamethasone-dependent increase of *Lyve1* expression. In contrast to *Lyve1*, but consistent with the in vivo findings, *CD44* expression remained unaltered by dexamethasone, PGE_2_, or the combination of both (Figure 2A, *lower panel*). 

To get further insights into how PGE_2_ might affect *Lyve1* expression, we next inhibited the four PGE_2_ receptors EP1-4 using specific antagonists 30 min prior to stimulation with PGE_2_ and dexamethasone. While inhibition of EP1, 3, and 4 did not affect the synergistic elevation of *Lyve1* by PGE_2_ and dexamethasone, EP2 antagonization completely abolished the PGE_2_-induced amplification of *Lyve1* (Figure 2B, *upper panel*). Again, *CD44* expression was neither affected by the stimuli nor the EP antagonists (Figure 2B, *lower panel*). Notably, while *EP1* and *2* were expressed at similar levels, *EP4* appeared to be expressed at a higher level, and *EP3* expression was almost undetectable in BMDM (Figure S1). Moreover, PGE_2_/dexamethasone stimulation slightly enhanced *EP2*, while it markedly reduced *EP4* expression.

The in vitro data support the enhancing effect of PGE_2_ for the HA receptor Lyve1 and exclude the same for CD44. Furthermore, they indicate that this effect is mediated via the EP2 receptor.

### 3.3. LMW-HA Enhances LPS-Induced TNF Expression in PGE_2_/Dexamethasone-Primed Macrophages

Lyve1 and CD44 are well-characterized HA receptors [16,28] and HA affects inflammatory processes. In detail, while low-molecular-weight-HA (LMW-HA) bears pro-inflammatory properties [29,30], high-molecular-weight-HA (HMW-HA) was previously shown to elicit anti-inflammatory effects [31,32]. Thus, we next asked if PGE_2_-dependent changes in Lyve1 expression might be functionally relevant in the context of HA-dependent inflammatory reactions. Therefore, we stimulated PGE_2_- and dexamethasone-primed macrophages with two different concentrations of LMW-HA (500 ng/mL or 500 µg/mL) or HMW-HA (500 µg/mL or 1000 µg/mL) to assess inflammation modulatory properties. Surprisingly, neither LMW-HA nor HMW-HA altered the expression of pro-inflammatory *tumor necrosis factor* (*Tnf*) irrespective of whether *Lyve1* expression was elevated by PGE_2_/dexamethasone priming or not (Figure S2A). In contrast, HMW-HA alone induced expression of anti-inflammatory *interleukin-10* (*Il10*) in BMDM, which was slightly enhanced after PGE_2_/dexamethasone priming (Figure S2B).

**Figure 3:**
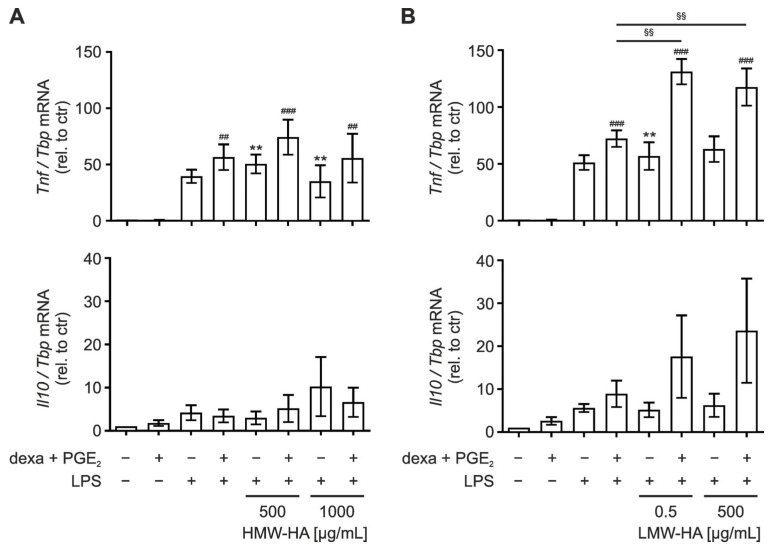
Hyaluronan amplifies LPS-induced inflammatory responses only in PGE_2_/dexamethasone-primed macrophages. Bone marrow-derived macrophages (BMDM) were differentiated for 5 days, primed for 48 h with dexamethasone (dexa; 100 ng/mL) and PGE_2_ (250 ng/mL), and treated for 1 h with high-molecular-weight hyaluronan (HMW-HA) (500 µg/mL or 1000 µg/mL) (**A**) or low-molecular-weight hyaluronan (LMW-HA) (500 ng/mL or 500 µg/mL) (**B**) prior to inflammatory stimulation with lipopolysaccharide (LPS; 100 ng/mL) for 1 h. mRNA expression of *Tnf* (*upper panels*) and *Il10* (*lower panels*) was determined by RT-qPCR analysis. Data are normalized to *Tbp* and presented relative to the untreated control as mean ± SEM (*n* = 5; * *p* < 0.05; ** *p* < 0.01; *** *p* < 0.001; compared to respective controls (* untreated, ^#^ dexa + PGE_2_, or ^§^ dexa + PGE_2_ + LPS stimulated).

Since the observed changes induced by LMW- or HMW-HA appeared rather minor, we next primed BMDM with PGE_2_/dexamethasone and LMW-HA or HMW-HA prior to stimulation with lipopolysaccharide (LPS, 100 ng/mL) for 1 h to elicit a substantial inflammatory response. LPS strongly induced the expression of *Tnf*, while it only slightly enhanced *Il10* expression (Figure 3A). PGE_2_/dexamethasone as well as HMW-HA priming alone or in combination neither affected LPS-induced *Tnf* nor *Il10* expression. Despite the fact that LMW-HA did not affect basal *Tnf* or *Il10* expression, it markedly boosted LPS-induced expression of *Tnf*, *Il10*, *Cxcl10*, and *Ifnβ*, yet only in PGE_2_/dexamethasone-primed cells (Figure 3B and Figure S3). Moreover, the boosting effect appeared more pronounced in those mRNAs already displaying stronger response to LPS (*Tnf* and *Cxcl10*). Noteworthy, this effect was already observed at a concentration of 0.5 µg/mL LMW-HA, suggesting that Lyve1 might be responsive even to minimal LMW-HA concentrations.

**Figure 4:**
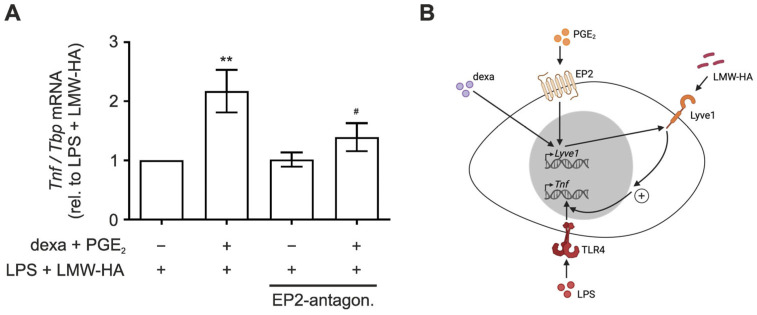
PGE_2_/dexamethasone priming sensitizes macrophages to enhanced inflammatory responses by hyaluronan via EP2 receptor. (**A**) Bone marrow-derived macrophages (BMDM) differentiated for 5 days, were pre-treated with the EP2 antagonist PF-04418948 (1 µM) for 30 min before priming for 48 h with dexamethasone (dexa; 100 ng/mL) and PGE_2_ (250 ng/mL). Then, the cells were treated for 1 h with low-molecular-weight hyaluronan (LMW-HA) (500 µg/mL) prior to inflammatory stimulation with lipopolysaccharide (LPS; 100 ng/mL) for 1 h. mRNA expression of *Tnf* was determined by RT-qPCR analysis. Data are normalized to *Tbp* and presented relative to LPS + LMW-HA-treated cells as mean ± SEM (*n* > 8; * *p* < 0.05; ** *p* < 0.01; compared to the respective LPS + LMW-HA-stimulated control (* no treatment, ^#^ dexa + PGE_2_ treatment)). (**B**) Schematic model of the proposed mechanism of PGE_2_-dependent aggravated inflammatory responses in macrophages by enhanced sensitization to LMW-HA via Lyve1.

Having established that PGE_2_ primes macrophages to elevated inflammatory responses upon LMW-HA stimulation, we aimed to gain further evidence for the role of the alternative HA receptor *Lyve1* in this context. To this end, we determined the effect of EP2 blockage on the LMW-HA-mediated increased inflammatory response to LPS in PGE_2_/dexamethasone-primed macrophages, as expression of the predominant HA-receptor CD44 was neither affected by PGE_2_ priming (Figure 2D) nor by the EP2 antagonist (Figure 2D). In line with the critical role of Lyve1 in this context, the LMW-HA-dependent increase in *Tnf* expression was completely blocked by the EP2 antagonist (Figure 4A), as were *Il10*, *Cxcl10* and *Ifnβ* expression (Figure S4).

Taken together, our data indicate that PGE_2_/dexamethasone stimulation enhances expression of the HA receptor Lyve1 in macrophages and sensitizes them towards LMW-HA-dependent augmentation of LPS-induced inflammatory responses.

## 4. Discussion

In this study, we explored regulation of hyaluronan receptor Lyve1 in macrophages in the course of inflammation. Specifically, we noticed that the inflammatory lipid mediator PGE_2_ adds to elevated Lyve1 expression in macrophages during the resolution phase in an in vivo peritonitis model. In vitro studies further suggested that PGE_2_ alone does not suffice to induce *Lyve1* expression, but rather synergizes with anti-inflammatory dexamethasone to enhance *Lyve1* levels. PGE_2_ signaling to increase *Lyve1* required an intact EP2 receptor. Functionally, PGE_2_/EP2-enforced *Lyve1* expression sensitized macrophages to pro-inflammatory activation by LMW-hyaluronan in the context of LPS stimulation (Figure 4B).

Macrophages play a crucial role during all phases of inflammation, i.e., while they take on a classical pro-inflammatory phenotype during early inflammation, their polarization changes in the course of inflammation to an alternatively activated wound healing and pro-resolving phenotype [15,33,34]. Polarization states underlie a strict regulation by auto- and paracrine factors within the inflammatory microenvironment. Noteworthy, selected bioactive lipid mediators affect macrophage polarization, and at the same time arise from macrophages to shape the inflammatory niche [35,36,37], often actually bearing pro- and anti-inflammatory properties. For example, prostaglandin E_2_, which is produced from arachidonic acid by the sequential activities of cyclooxygenases 1 or 2 and specific prostaglandin E_2_ synthases, i.e., mPGES-1 and cPGES, is well characterized as a pro-inflammatory mediator eliciting both local as well as systemic effects after an inflammatory stimulation [38,39]. We recently also identified a pro-resolving function of PGE_2_, which was independent from its impact on the establishment of the inflammation [26]. Specifically, we found that PGE_2_ contributed to resolution of inflammation by preventing CX3CL1-mediated retention of activated myeloid cells at sites of injury. In the same model, expression of the hyaluronan receptor *Lyve1* was reduced upon inhibition of mPGES-1. Thus, in contrast to the CX3CL1/CX3CR1 axis, *Lyve1* levels in macrophages negatively correlated with PGE_2_ levels in the course of peritonitis, indicating that Lyve1 might have a pro-resolving function. This assumption was further supported by the kinetics of Lyve1 expression, which coincided with the resolving activity. Specifically, in the absence of a PGE_2_-targeted intervention the expression of *Lyve1* peaked three days after zymosan treatment, when resolution processes are expected to be most active.

While low-molecular-weight fragments (LMW) of hyaluronan (hyaluronic acid, HA) have been shown to elicit pro-inflammatory signals [40,41,42], high-molecular-weight-HA was reported to induce anti-inflammatory responses [18,20,31]. Despite the fact that Lyve1 is well characterized as a receptor for HA [16], its role in inflammation remains obscure. In line with the proposed anti-inflammatory properties of HMW-HA, we observed elevated *Il10* expression in macrophages upon stimulation with HMW-HA alone. Though this effect was only slightly augmented by enhanced *Lyve1* expression upon PGE_2_/dexamethasone priming. Thus, Lyve1 likely does not play a major role in transmitting the anti-inflammatory activity of HMW-HA. Unexpectedly, LMW-HA alone neither altered the expression of pro-inflammatory *Tnf* nor of anti-inflammatory *Il10*, irrespective of Lyve1 expression. Interestingly, additional priming of PGE_2_/dexametasone-treated macrophages with LMW-HA substantially increased inflammatory responses to subsequent stimulation with LPS. As neither PGE_2_/dexamethasone nor LMW-HA alone altered the LPS response, these findings indicate that PGE_2_/dexamethasone and LMW-HA synergistically sensitize macrophages to inflammatory activation. Since inhibition of the PGE_2_ receptor EP2 effectively prevented not only PGE_2_/dexamethasone-mediated *Lyve1* expression but also the synergistic LPS-sensitizing potential of combined PGE_2_/dexamethasone and LMW-HA priming, we propose that the PGE_2_/EP2-dependent increase in Lyve1 allows for binding of LMW-HA, which in turn promotes LPS-induced pro-inflammatory responses. Previously, the pro-inflammatory activity of LMW-HA was attributed to its interaction with either CD44 or Toll-like receptors (TLR), including TLR2 and TLR4, consequently activating MAPK signaling and/or transcription factor nuclear factor κB (NFκB) [43,44]. These findings indicate that matrix remodeling not only affects the ability of lymphocytes to infiltrate into inflamed tissues but, at the same time, extracellular matrix degradation products contribute to the formation of the inflammatory environment. Lyve1 appeared of specific relevance to integrating matrix-derived environmental signals to tune inflammatory responses. Along these lines, Lyve1-positive macrophages were proposed to exert pro-angiogenic functions and further seem to play an important role in inflammatory conditions like rheumatoid arthritis [45,46]. In addition, the absence of Lyve1-positive macrophages was associated with impaired lung and heart fibrosis [9]. Notably, the well-characterized hyaluronan receptor CD44 was not altered in this context, whereas it was previously shown to display anti-inflammatory properties and the knockout of CD44 in macrophages was associated with higher levels of pro-inflammatory cytokines in a peritonitis model [47,48]. Thus, it appears relevant to consider the entire spectrum of HA receptors in inflammatory conditions, as they might counteract each other or alternatively compete for HA [20,49]. The observation that even low concentrations of LMW-HA sufficed to induce the synergistic inflammatory response in Lyve1-expressing macrophages indicates that Lyve1 might act as a sensor for the presence of LMW-HA.

As a side note, the observation that dexamethasone synergizes with PGE_2_ to induce Lyve1 and thereby contributes to sensitization to inflammatory stimulation corroborates previous findings suggesting that glucocorticoids foster some inflammatory responses [27]. It would be of great interest to see if and identify which endogenous glucocorticoids might contribute to the increase in Lyve1-expressing macrophages during the resolution of inflammation. Furthermore, it remains to be shown whether the presence of Lyve1-positive macrophages during the resolution of inflammation indeed counterbalances anti-inflammatory responses or instead supports resolution by an alternative mechanism.

## 5. Conclusions

Identification of the PGE_2_/EP2-elicited increase in hyaluronan receptor Lyve1 expression in the course of inflammation, and its supporting function in LPS-mediated inflammatory responses, provides insights into a still under-investigated aspect of the inflammatory niche, i.e., the impact of matrix-derived factors on the course of inflammation. Furthermore, characterization of the regulatory principle of Lyve1 might allow for the future development of resolution-modulatory intervention strategies targeting Lyve1-dependent increases in inflammatory responses.

## Figures and Tables

**Table 1:** Primers for qPCR.

Target	Forward	Reverse
*CD44*	5′-ACG AGG AGG AGG TGT GAT GT-3′	5′-TCG CTT GTG AAA GCA CCA AC-3′
*TBP*	5′-CTG ACC ACT GCA CCG TTG CCA-3′	5′-GAC TGC AGC AAA TCG CTT GGG A-3′
*TNF*	5′-CTG AAC TTC GGG GTG ATC GG-3′	5′-GGC TTG TCA CTC GAA TTT TGA GA-3′
*EP1*	5′-CAT GGT CTT CTT CGG CCT GT-3′	5′-GAT CAG TGG CTG CGT GAC A-3′
*EP2*	5′-GGA GAC GGA CCA CCT CAT TC-3′	5′-TCC ATG TAG GCA AAG ATT GTG AA-3′
*EP3*	5′-TAA TTG CAG TTC GCC TGG CT-3′	5′-GGT TGT TCA TCA TCT GGC AGA AC-3′
*EP4*	5′-ACC TGA CTG AAA GCA GCC TC-3′	5′-AAG TTC TCA GCG AGG TGG TG-3′
*IL10*	5′-GCT CTT ACT GAC TGG CAT GAG-3′	5′-CGC AGC TCT AGG AGC ATG TG-3′
*LYVE1*	5′-CAG CAC ACT AGC CTG GTG TTA-3′	5′-CGC CCA TGA TTC TGC ATG TAG A-3′

## Data Availability

Data are contained within the article and Supplementary Materials.

## Supplementary Materials

The following supporting information can be downloaded at: https://www.mdpi.com/article/10.3390/biology12111441/s1, Figure S1: Differential expression of PGE_2_ receptors, Figure S2: Effect of hyaluronan on the expression of inflammatory mediators, Figure S3: LMW-HA amplifies LPS-induced inflammatory responses in PGE_2_/dexamethasone-primed macrophages, Figure S4: PGE_2_/dexamethasone priming sensitizes macrophages to enhanced inflammatory responses by LMW-HA via EP2 receptor.

## References

[1] Jentho E., Weis S. (2021). DAMPs and Innate Immune Training. Front. Immunol..

[2] Medzhitov R. (2008). Origin and Physiological Roles of Inflammation. Nature.

[3] Schett G., Neurath M.F. (2018). Resolution of Chronic Inflammatory Disease: Universal and Tissue-Specific Concepts. Nat. Commun..

[4] Sindrilaru A., Peters T., Wieschalka S., Baican C., Baican A., Peter H., Hainzl A., Schatz S., Qi Y., Schlecht A. (2011). An Unrestrained Proinflammatory M1 Macrophage Population Induced by Iron Impairs Wound Healing in Humans and Mice. J. Clin. Investig..

[5] Swirski F.K., Pittet M.J., Kircher M.F., Aikawa E., Jaffer F.A., Libby P., Weissleder R. (2006). Monocyte Accumulation in Mouse Atherogenesis Is Progressive and Proportional to Extent of Disease. Proc. Natl. Acad. Sci. USA.

[6] Kasikara C., Doran A.C., Cai B., Tabas I. (2018). The Role of Non-Resolving Inflammation in Atherosclerosis. J. Clin. Investig..

[7] Newson J., Stables M., Karra E., Arce-Vargas F., Quezada S., Motwani M., Mack M., Yona S., Audzevich T., Gilroy D.W. (2014). Resolution of Acute Inflammation Bridges the Gap between Innate and Adaptive Immunity. Blood.

[8] Mills C.D., Kincaid K., Alt J.M., Heilman M.J., Hill A.M. (2000). M-1/M-2 Macrophages and the Th1/Th2 Paradigm. J. Immunol..

[9] Chakarov S., Lim H.Y., Tan L., Lim S.Y., See P., Lum J., Zhang X.-M., Foo S., Nakamizo S., Duan K. (2019). Two Distinct Interstitial Macrophage Populations Coexist across Tissues in Specific Subtissular Niches. Science.

[10] Mantovani A., Sica A., Sozzani S., Allavena P., Vecchi A., Locati M. (2004). The Chemokine System in Diverse Forms of Macrophage Activation and Polarization. Trends Immunol..

[11] Smigiel K.S., Parks W.C. (2018). Macrophages, Wound Healing, and Fibrosis: Recent Insights. Curr. Rheumatol. Rep..

[12] Zaynagetdinov R., Sherrill T.P., Kendall P.L., Segal B.H., Weller K.P., Tighe R.M., Blackwell T.S. (2013). Identification of Myeloid Cell Subsets in Murine Lungs Using Flow Cytometry. Am. J. Respir. Cell Mol. Biol..

[13] Lakschevitz F.S., Hassanpour S., Rubin A., Fine N., Sun C., Glogauer M. (2016). Identification of Neutrophil Surface Marker Changes in Health and Inflammation Using High-Throughput Screening Flow Cytometry. Exp. Cell Res..

[14] Blériot C., Chakarov S., Ginhoux F. (2020). Determinants of Resident Tissue Macrophage Identity and Function. Immunity.

[15] Jablonski K.A., Amici S.A., Webb L.M., Ruiz-Rosado J.D.D., Popovich P.G., Partida-Sanchez S., Guerau-de-Arellano M. (2015). Novel Markers to Delineate Murine M1 and M2 Macrophages. PLoS ONE.

[16] Banerji S., Ni J., Wang S.-X., Clasper S., Su J., Tammi R., Jones M., Jackson D.G. (1999). LYVE-1, a New Homologue of the CD44 Glycoprotein, Is a Lymph-Specific Receptor for Hyaluronan. J. Cell Biol..

[17] Jiang D., Liang J., Noble P.W. (2007). Hyaluronan in Tissue Injury and Repair. Annu. Rev. Cell Dev. Biol..

[18] Ruppert S.M., Hawn T.R., Arrigoni A., Wight T.N., Bollyky P.L. (2014). Tissue Integrity Signals Communicated by High-Molecular Weight Hyaluronan and the Resolution of Inflammation. Immunol. Res..

[19] Baeva L.F., Lyle D.B., Rios M., Langone J.J., Lightfoote M.M. (2014). Different Molecular Weight Hyaluronic Acid Effects on Human Macrophage Interleukin 1β Production: Effects of Lmw+Ha on Human Monocytes. J. Biomed. Mater. Res..

[20] Cyphert J.M., Trempus C.S., Garantziotis S. (2015). Size Matters: Molecular Weight Specificity of Hyaluronan Effects in Cell Biology. Int. J. Cell Biol..

[21] Zhang G., Gao Y., Zhao Z., Pyykko I., Zou J. (2023). Low-Molecular-Weight Hyaluronic Acid Contributes to Noise-Induced Cochlear Inflammation. Audiol. Neurootol..

[22] Dick S.A., Wong A., Hamidzada H., Nejat S., Nechanitzky R., Vohra S., Mueller B., Zaman R., Kantores C., Aronoff L. (2022). Three Tissue Resident Macrophage Subsets Coexist across Organs with Conserved Origins and Life Cycles. Sci. Immunol..

[23] Wang Y., Chaffee T.S., LaRue R.S., Huggins D.N., Witschen P.M., Ibrahim A.M., Nelson A.C., Machado H.L., Schwertfeger K.L. (2020). Tissue-Resident Macrophages Promote Extracellular Matrix Homeostasis in the Mammary Gland Stroma of Nulliparous Mice. eLife.

[24] Anstee J.E., Feehan K.T., Opzoomer J.W., Dean I., Muller H.P., Bahri M., Cheung T.S., Liakath-Ali K., Liu Z., Choy D. (2023). LYVE-1+ Macrophages Form a Collaborative CCR5-Dependent Perivascular Niche That Influences Chemotherapy Responses in Murine Breast Cancer. Dev. Cell.

[25] Slysz J., Sinha A., DeBerge M., Singh S., Avgousti H., Lee I., Glinton K., Nagasaka R., Dalal P., Alexandria S. (2023). Single-Cell Profiling Reveals Inflammatory Polarization of Human Carotid versus Femoral Plaque Leukocytes. JCI Insight.

[26] Rappl P., Rösser S., Maul P., Bauer R., Huard A., Schreiber Y., Thomas D., Geisslinger G., Jakobsson P.-J., Weigert A. (2021). Inhibition of MPGES-1 Attenuates Efficient Resolution of Acute Inflammation by Enhancing CX3CL1 Expression. Cell Death Dis..

[27] Dollt C., Becker K., Michel J., Melchers S., Weis C.-A., Schledzewski K., Krewer A., Kloss L., Gebhardt C., Utikal J. (2017). The Shedded Ectodomain of Lyve-1 Expressed on M2-like Tumor-Associated Macrophages Inhibits Melanoma Cell Proliferation. Oncotarget.

[28] Peach R., Hollenbaugh D., Stamenkovic I., Aruffo A. (1993). Identification of Hyaluronic Acid Binding Sites in the Extracellular Domain of CD44. J. Cell Biol..

[29] Noble P.W., McKee C.M., Cowman M., Shin H.S. (1996). Hyaluronan Fragments Activate an NF-Kappa B/I-Kappa B Alpha Autoregulatory Loop in Murine Macrophages. J. Exp. Med..

[30] Collins S.L., Black K.E., Chan-Li Y., Ahn Y.-H., Cole P.A., Powell J.D., Horton M.R. (2011). Hyaluronan Fragments Promote Inflammation by Down-Regulating the Anti-Inflammatory A2a Receptor. Am. J. Respir. Cell Mol. Biol..

[31] Shi Q., Zhao L., Xu C., Zhang L., Zhao H. (2019). High Molecular Weight Hyaluronan Suppresses Macrophage M1 Polarization and Enhances IL-10 Production in PM2.5-Induced Lung Inflammation. Molecules.

[32] Bonet I.J.M., Khomula E.V., Araldi D., Green P.G., Levine J.D. (2021). PI3Kγ/AKT Signaling in High Molecular Weight Hyaluronan (HMWH)-Induced Anti-Hyperalgesia and Reversal of Nociceptor Sensitization. J. Neurosci..

[33] Shapouri-Moghaddam A., Mohammadian S., Vazini H., Taghadosi M., Esmaeili S., Mardani F., Seifi B., Mohammadi A., Afshari J.T., Sahebkar A. (2018). Macrophage Plasticity, Polarization, and Function in Health and Disease. J. Cell. Physiol..

[34] Xue J., Schmidt S.V., Sander J., Draffehn A., Krebs W., Quester I., De Nardo D., Gohel T.D., Emde M., Schmidleithner L. (2014). Transcriptome-Based Network Analysis Reveals a Spectrum Model of Human Macrophage Activation. Immunity.

[35] Giannakis N., Sansbury B.E., Patsalos A., Hays T.T., Riley C.O., Han X., Spite M., Nagy L. (2019). Dynamic Changes to Lipid Mediators Support Transitions among Macrophage Subtypes during Muscle Regeneration. Nat. Immunol..

[36] Van Dierendonck X.A.M.H., Vrieling F., Smeehuijzen L., Deng L., Boogaard J.P., Croes C.-A., Temmerman L., Wetzels S., Biessen E., Kersten S. (2022). Triglyceride Breakdown from Lipid Droplets Regulates the Inflammatory Response in Macrophages. Proc. Natl. Acad. Sci. USA.

[37] MacKenzie K.F., Clark K., Naqvi S., McGuire V.A., Nöehren G., Kristariyanto Y., Van Den Bosch M., Mudaliar M., McCarthy P.C., Pattison M.J. (2013). PGE2 Induces Macrophage IL-10 Production and a Regulatory-like Phenotype via a Protein Kinase A–SIK–CRTC3 Pathway. J. Immunol..

[38] Nakanishi M., Rosenberg D.W. (2013). Multifaceted Roles of PGE2 in Inflammation and Cancer. Semin. Immunopathol..

[39] Kalinski P. (2012). Regulation of Immune Responses by Prostaglandin E2. J. Immunol..

[40] McKee C.M., Penno M.B., Cowman M., Burdick M.D., Strieter R.M., Bao C., Noble P.W. (1996). Hyaluronan (HA) Fragments Induce Chemokine Gene Expression in Alveolar Macrophages. The Role of HA Size and CD44. J. Clin. Investig..

[41] Petrey A.C., de la Motte C.A. (2014). Hyaluronan, a Crucial Regulator of Inflammation. Front. Immunol..

[42] McKee C.M., Lowenstein C.J., Horton M.R., Wu J., Bao C., Chin B.Y., Choi A.M.K., Noble P.W. (1997). Hyaluronan Fragments Induce Nitric-Oxide Synthase in Murine Macrophages through a Nuclear Factor ΚB-Dependent Mechanism. J. Biol. Chem..

[43] Yamawaki H., Hirohata S., Miyoshi T., Takahashi K., Ogawa H., Shinohata R., Demircan K., Kusachi S., Yamamoto K., Ninomiya Y. (2008). Hyaluronan Receptors Involved in Cytokine Induction in Monocytes. Glycobiology.

[44] Jiang D., Liang J., Noble P.W. (2011). Hyaluronan as an Immune Regulator in Human Diseases. Physiol. Rev..

[45] Alivernini S., MacDonald L., Elmesmari A., Finlay S., Tolusso B., Gigante M.R., Petricca L., Di Mario C., Bui L., Perniola S. (2020). Distinct Synovial Tissue Macrophage Subsets Regulate Inflammation and Remission in Rheumatoid Arthritis. Nat. Med..

[46] Kieu T.Q., Tazawa K., Kawashima N., Noda S., Fujii M., Nara K., Hashimoto K., Han P., Okiji T. (2022). Kinetics of LYVE-1-Positive M2-like Macrophages in Developing and Repairing Dental Pulp in Vivo and Their pro-Angiogenic Activity in Vitro. Sci. Rep..

[47] Van Der Windt G.J.W., Van ′T Veer C., Florquin S., Van Der Poll T. (2010). CD44 Deficiency Is Associated with Enhanced *Escherichia Coli* -Induced Proinflammatory Cytokine and Chemokine Release by Peritoneal Macrophages. Infect. Immun..

[48] Qadri M., Almadani S., Jay G.D., Elsaid K.A. (2018). Role of CD44 in Regulating TLR2 Activation of Human Macrophages and Downstream Expression of Proinflammatory Cytokines. J. Immunol..

[49] Johnson L.A., Jackson D.G. (2021). Hyaluronan and Its Receptors: Key Mediators of Immune Cell Entry and Trafficking in the Lymphatic System. Cells.

