# Supplementary material for: Prostaglandin E2 Boosts the Hyaluronan-Mediated Increase in Inflammatory Response to Lipopolysaccharide by Enhancing Lyve1 Expression
Source: Biology (Basel). 2023 Nov 16;12(11):1441. doi: 10.3390/biology12111441 (PMC10669677; doi:10.3390/biology12111441)
Supplement: Supplementary file 1 [file biology-12-01441-s001.zip › biology-2700417-supplementary.pdf]

Prostaglandin E2 Boosts the Hyaluronan-Mediated Increase in Inflammatory Response to Lipopolysaccharide by Enhancing Lyve1 Expression

Pauline Hog, Silvia Kuntschar, Peter Rappl, Arnaud Huard, Andreas Weigert, Bernhard Brüne, Tobias Schmid

SUPPLEMENTARY FIGURES

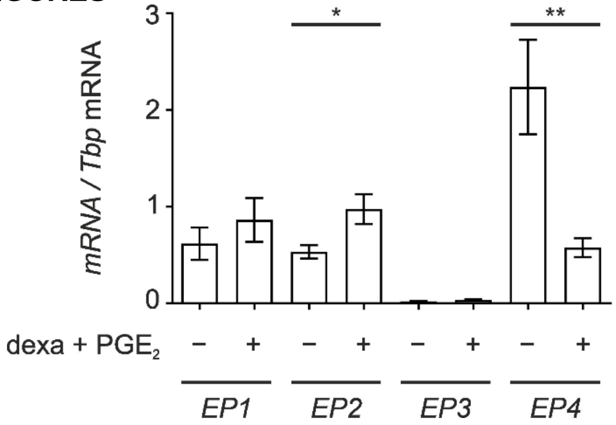

Figure S1. Differential expression of PGE_2 receptors. Bone marrow-derived macrophages (BMDM) were differentiated for 5 days before stimulation for 48 h with dexamethasone (dexa; 100 ng/mL) and PGE_2 (250 ng/mL). Expression of PGE_2 receptors *EP1*, *EP2*, *EP3*, and *EP4* was determined by RT-qPCR analysis, normalized to *Tbp,* and statistical differences were evaluated using two-tailed paired student's t-test. Data are presented as mean ± SEM (n = 13; * $p < 0.05$; ** $p < 0.01$).

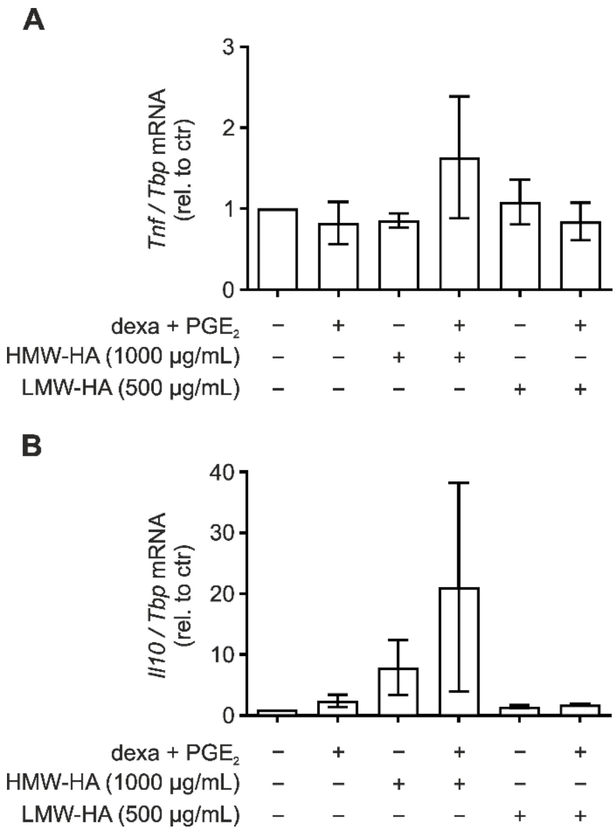

Figure S2. Effect of hyaluronan on the expression of inflammatory mediators. Bone marrow-derived macrophages (BMDM) were differentiated for 5 days, primed for 48 h with dexamethasone (dexa; 100 ng/mL) and PGE_2 (250 ng/mL), and treated for 1 h with high molecular weight hyaluronan (HMW-HA) (1,000 µg/mL) or low molecular weight hyaluronan (LMW-HA) (500 µg/mL). mRNA expression of *Tnf* (**A**) and *Il10* (**B**) was determined by RT-qPCR analysis, normalized to *Tbp*, and statistical differences were evaluated using one-way ANOVA with Tukey's posthoc test. Data are presented as mean ± SEM (*n* = 3).

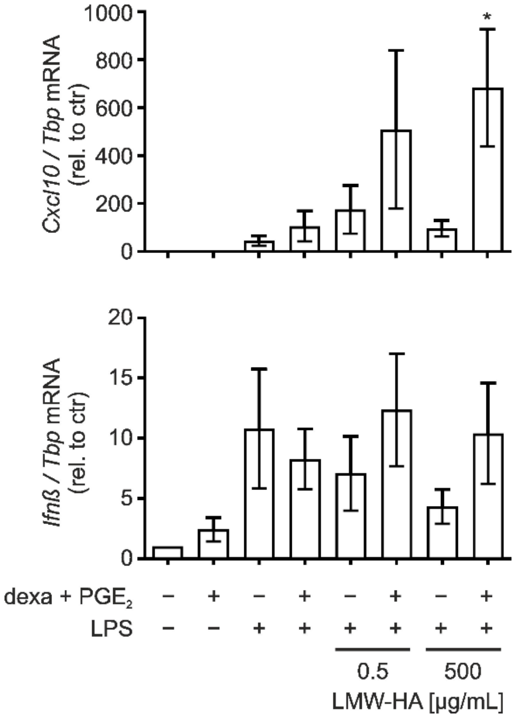

Figure S3. LMW-HA amplifies LPS-induced inflammatory responses in PGE_2/dexamethasone-primed macrophages. Bone marrow-derived macrophages (BMDM) were differentiated for 5 days, primed for 48 h with dexamethasone (dexa; 100 ng/mL) and PGE_2 (250 ng/mL), and treated for 1 h with low molecular weight hyaluronan (LMW-HA) (500 ng/mL or 500 µg/mL) prior to inflammatory stimulation with lipopolysaccharide (LPS; 100 ng/mL) for 1 h. mRNA expression of *Cxcl10* (*upper panel*) and *IFNβ* (*lower panel*) was determined by RT-qPCR analysis. Data are normalized to *Tbp* and presented relative to untreated control as mean ± SEM ($n = 5$; * $p < 0.05$; compared to untreated control).

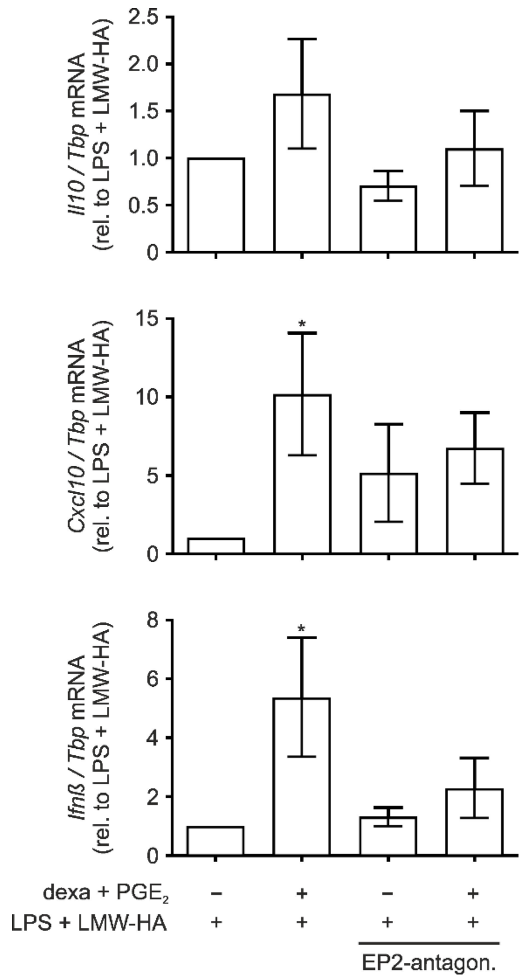

Figure S4. PGE_2/dexamethasone priming sensitizes macrophages to enhanced inflammatory responses by LMW-HA via EP2 receptor. Bone marrow-derived macrophages (BMDM) differentiated for 5 days, were pre-treated with the EP2 antagonist PF-04418948 (1 µM) for 30 min before priming for 48 h with dexamethasone (dexa; 100 ng/mL) and PGE_2 (250 ng/mL). Then the cells were treated for 1 h with low molecular weight hyaluronan (LMW-HA) (500 µg/mL) prior to inflammatory stimulation with lipopolysaccharide (LPS; 100 ng/mL) for 1 h. mRNA expression of *Il10* (*upper panel*), *Cxcl10* (*middle panel*), and *Ifnβ* (*lower panel*) was determined by RT-qPCR analysis. Data are normalized to *Tbp* and presented relative to LPS + LMW-HA-treated cells as mean ± SEM ($n > 7$; * $p < 0.05$; compared to LPS + LMW-HA-treated control).
